# Improved Lipid Profile Associated with Daily Consumption of Tri-Sura-Phon in Healthy Overweight Volunteers: An Open-Label, Randomized Controlled Trial

**DOI:** 10.1155/2017/2687173

**Published:** 2017-04-06

**Authors:** Sirigoon Kuamsub, Pariyaphat Singthong, Wipawee Chanthasri, Nicharee Chobngam, Warissara Sangkaew, Sasithorn Hemdecho, Thammarat Kaewmanee, Sasitorn Chusri

**Affiliations:** ^1^Natural Product Research Center of Excellence and Faculty of Traditional Thai Medicine, Prince of Songkla University, Hat Yai, Songkhla 90110, Thailand; ^2^Faculty of Thai Traditional and Alternative Medicine, Ubonratchathani Rajabhat University, Meung, Ubonratchathani 34000, Thailand; ^3^Sirindhorn College of Public Health Trang, Kantang, Trang 92110, Thailand; ^4^Department of Food Science and Nutrition, Faculty of Science and Technology, Prince of Songkla University, Pattani 94000, Thailand

## Abstract

Tri-Sura-Phon (TSP), a traditional Thai polyherbal formula renowned for its rejuvenating properties, is commonly used as a blood tonic. It comprises* Cinnamomum bejolghota*,* Cinnamomum parthenoxylon*, and* Aquilaria crassna*. The aim of this study is to evaluate the beneficial properties of TSP tea consumption on blood glucose regulation and serum lipid profiles of healthy overweight volunteers. This open-label, randomized controlled trial was conducted in 70 healthy overweight adults. Two groups of 35 subjects took a TSP infusion or a placebo (cornstarch) twice daily for 8 weeks. The blood glucose regulation, serum lipid profiles, BMI, and liver function tests of the subjects were determined at the baseline, 4th week, and endpoint (8th week). Significant decreases in the average fasting levels of total cholesterol (*p* = 0.013), triglyceride (*p* = 0.001), and low-density lipoprotein (LDL, *p* = 0.017) were observed in the TSP group at the 8th week compared to those at the baseline. The average HDL level in the TSP group at the beginning of the study was 65.2 mg/dL, and it increased significantly (*p* = 0.005) to 72.4 mg/dL after 8 weeks of TSP intake. This study showed that the intake of TSP tea as an antioxidant-rich beverage might be safe and improve lipid profiles in overweight adults.

## 1. Introduction

Obesity has become a major global health concern because it is directly related to the incidence of type 2 diabetes [1], hypertension, hypercholesterolemia [2], acceleration of aging process [3], and arteriosclerosis. In addition, obesity is associated with significant increases in oxidative stress owing to the well-established relationship between oxidative stress and metabolic syndrome [4]. In vivo studies have clearly demonstrated that treating obese animal models with antioxidant agents attenuates the development of hyperlipidemia and diabetes [5, 6]. In recent years, there has been a growing interest in natural bioactive compounds (especially plant-derived antioxidants) that may protect humans from the aforementioned diseases. Some traditional medicinal plants that possess antioxidant activity and contain high amounts of phenolics are green tea* (Camellia sinensis)* [7],* Salvia officinalis* [8],* Melissa officinalis* [9], and* Capparis spinosa* [10]. These plants have been clinically proven to reduce the development of hyperlipidaemia and type 2 diabetes. Although intensive studies have been performed on these medicinal plants, very few studies have reported the potential benefits of antioxidant-rich herbal formulations described in traditional medicine. Studies have been extensively performed on* Triphala*, a polyherbal formula made from the fruits of* Terminalia chebula*,* Terminalia bellirica*, and* Phyllanthus emblica*. It has been widely described as a rejuvenator in Ayurvedic medicine.* Triphala *has notable health benefits as an effective antioxidant, chemopreventive, radioprotective, antidiabetic, and hypolipidemic agent both in vitro and in vivo [11, 12]. It has also been clinically proven to be a promising antiplaque and antigingivitis agent [13].

According to the Thai Pharmaceutical Textbook,* Tri-Sura-Phon* (TSP) is a simple formulation that comprises a mixture of powdered dried wood from* Aquilaria crassna* and* Cinnamomum parthenoxylon*, as well as the bark of* Cinnamomum bejolghota*. In Thai,* Tri-Sura- Phon* means three* (Tri*-) medicinal plants that have potent* (Sura*-) health benefits* (Phon)* [14]. An earlier study conducted in our laboratory revealed that, among the 18 tested rejuvenative recipes used in traditional Thai medicine, TSP exhibits remarkable activity as DPPH (2,2-diphenyl-1-picrylhydrazyl) and ABTS (2,2′-azino-bis(3-ethylbenzthiazoline-6-sulphonic acid)) free radical scavengers and produces no cytotoxic effects on* Vero* cells [15]. Moreover, its herbal components have been credited with diverse beneficial properties such as antioxidant, anti-ischemic, antimicrobial, anticancer, hypoglycaemic, and hypolipidemic activities [16–18]. Besides this broad spectrum of biological activities, no toxic effects were observed at doses as high as 2000, 300, and 2500 mg/kg of* A. crassna* [18],* C. parthenoxylon* [16], and* C. bejolghota *[17], respectively, in in vivo studies.

Although* Tri-Sura-Phon* is traditionally used as a rejuvenating agent and blood tonic, to our knowledge, no clinical studies have been conducted to determine the beneficial effects of TSP consumption. This single centre, open-label, randomized controlled study was designed to evaluate the possible beneficial health effects of TSP tea consumption on fasting blood glucose (FBG) levels, insulin levels, lipid profiles, and liver functions in humans, and to compare these effects with those of a placebo. A pilot trial with healthy overweight volunteers had demonstrated that TSP tea consumption significantly improved blood lipid profiles without adverse effects. This information paves the way for future studies designed to test the hypolipidemic potential of TSP in patients with borderline hyperlipidemia.

## 2. Material and Methods

### 2.1. Medicinal Plants

#### 2.1.1. Plant Materials

**Figure 1:**
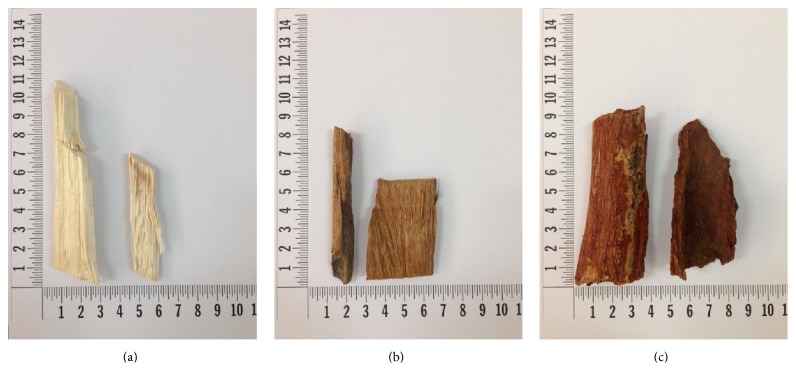
A polyherbal formulation,* Tri-Sura-Phon*, contains equal parts of* Aquilaria crassna* (a),* Cinnamomum parthenoxylon* (b), and* Cinnamomum bejolghota* (c).

*C. bejolghota *(bark),* A. crassna* (wood), and* C. parthenoxylon* (wood) were obtained from the licensed traditional medical drug store, Triburi Orsot, in Songkla, Thailand (Figure 1). Voucher specimens of crude drugs (*C. bejolghota*; THP-R0051,* A. crassna*; THP-R0052, and* C. parthenoxylon*; THP-R0053) were deposited in the herbarium within the Faculty of Traditional Thai Medicine, Prince of Songkla University. The plant parts were cleaned, dried at 60°C for 72 h in an air blowing thermostatic oven, and stored in air tight containers at 4°C until further use. The plant materials were independently milled into powder, sieved, weighed (1 : 1 : 1) in equal proportions, and then mixed to obtain* Tri-Sura-Phon* (TSP) as per the procedure described in the Thai Pharmaceutical Textbook [14].

#### 2.1.2. Standardization of Medicinal Plants

The tested medicinal plants and TSP were standardized using their total phenolic and flavonoid contents, which were measured by the Folin-Ciocalteu assay and aluminium chloride reaction, respectively [19].* Tri-Sura-Phon* (500 g) was submitted for hydrodistillation using a Clevenger-type apparatus at 100°C for 5 h. Gas chromatography (GC) analysis was carried out using an Agilent 7890A GC System equipped with a flame ionization detector (FID). A TRACE™ TR-1MS fused-silica capillary column (30 m × 0.32 mm id, film thickness 0.25 *μ*m; Thermo Scientific, San Jose, CA) was employed. The operating conditions were as follows: initial oven temperature, 60°C held for 2 min, then gradually increased to 250°C at 4°C/min, and held for 20 min; injector and detector temperatures, 250°C; carrier gas, 1.0 mL/min helium; injection volume, 1 *μ*L; split ratio, 10 : 1. Quantitative data were compared with authentic active constituents including 1, 8-cineole, alpha-terpineol, linalool, and methyl eugenol, which were purchased from Sigma-Aldrich (St. Louis, MO).

### 2.2. Preparation of TSP and Placebo

Teabags (each containing 1.25 g of TSP powder) were manufactured at the Traditional Thai Medicine Hospital, Prince of Songkla University, Hat Yai Thailand, specifically for the present study. TSP teas were brewed by the addition of one teabag per 100 mL of freshly boiled water at 95°C for 1, 3, and 5 min without stirring. Each teabag was squeezed with a spoon and removed from the water before analysis. The antioxidant capacities of the teabags at a concentration of 1% v/v were determined as mentioned above. Sensory responses for colour, odour, taste, and overall acceptability were evaluated by 30 trained panellists using a 9-point hedonic scale (1 dislike very much; 5 neither like nor dislike; 9 like very much) [20]. The free radical scavenging activities were determined according to the elimination of DPPH and ABTS radicals [19].

The packaging of TSP tea and placebo (cornstarch) were identical, and they were sealed in opaque sterilized bags and placed inside a cardboard box. All boxes were identified by the letter “A” for placebo or “B” for TSP. To ensure the consistency of tea preparation, standard 100 mL ceramic cups with lids and specific written instructions for infusing the teabag with boiling water (without sugar or other sweetening agents) in the cup for 3 min before removing the teabag were included in the cardboard box. The identification of the box was known only by a licensed Thai traditional pharmacist who was not involved in data collection or analyses. The participants, investigators, and data gatherers were masked from the intervention procedures.

### 2.3. Study Design

This study was conducted from July 2013 to March 2014 at the Traditional Thai Medical Hospital, Prince of Songkla University in Hat Yai, Thailand. Considering *α* = 0.05, power of the test = 80%, and achieving a 13 mg/dL difference in total cholesterol between the groups, the sample size was estimated to be 29. Assuming 20% attrition, 35 participants in each group were included. The inclusion criteria for this study were as follows: (1) men or women between 20 and 55 years, (2) body mass index (BMI) of 23–29.9 kg/m^2^ [21], (3) being in good health (patients with no medical histories of severe health problems such as hypertension, cardiovascular disease, dyslipidemia, clinical depression, diabetes mellitus, and thyroid diseases and no symptoms and abnormal findings during both physical examination and routine laboratory tests), and (4) willingness to fill out the questionnaires for this trial. Participants with the following conditions were excluded: (1) taking medication or supplements that might have an effect on metabolism or appetite and/or use of weight control therapy or antioxidant products within the last 3 months, (2) having a history of allergic reactions to medications or food, (3) being medically controlled or diagnosed with severe health problems such as hypertension, cardiovascular disease, dyslipidemia, clinical depression, diabetes mellitus, and thyroid diseases, and (4) being currently pregnant or lactating or both.

Letters were sent to 250 local participants who live near (less than 5 km) the university to explain the purpose of the study and invite them to participate. A detailed explanation of the study design was given to the volunteers who expressed willingness to participate in the study. Written informed consent was obtained from the participants at the initiation of the study. All participants were free to withdraw at any time during the course of the study. The protocol was approved by the Medical Ethics Committee of Faculty of Traditional Thai Medicine, Prince of Songkla University (approval number: EC.56/B 06-001).

### 2.4. Interventions

All participants were randomly assigned to either the experimental (TSP) or control group (placebo) throughout this period. A random number between A0.01 and A0.70 was generated by the computer for each participant. The participants with a random number between A0.01 and A0.35 were assigned to the TSP group, while those with a random number between A0.36 and A0.70 were assigned to the placebo group. The eligible participants were assigned to receive either TSP tea or placebo twice daily, 20 min before breakfast and dinner, for 8 consecutive weeks. They received detailed advice on diet, exercise, and lifestyle modification as indicated in the 2013 AHA/ACC/TOS Guideline for the Management of Overweight and Obesity in Adults [22]. Patient compliance with the allocated treatments was monitored by filling a daily compliance chart. In addition, the compliance was measured by counting the product usage at weeks 4 and 8.

### 2.5. Measurements

All measurements were done after fasting for 12 h and determined at the baseline, 4 weeks, and endpoint (8th week) of the study for both groups. For the assessment of herbal drug efficacy or any side effects due to the treatment, insulin levels were measured by an electrochemiluminescence immunoassay (ECLIA-Roche Diagnostics GmbH, D-68298 Mannheim). The levels of FBG, cholesterol, low-density lipoprotein (LDL), high-density lipoprotein (HDL), triglyceride, albumin, total bilirubin, direct bilirubin, aspartate aminotransferase (AST), alanine aminotransferase (ALT), alkaline phosphatase (ALP), and gamma-glutamyl transpeptidase (GGT) were determined with standard enzymatic colorimetric techniques and measured using the Hitachi 902 automatic analyser (Hitachi, Japan). In addition, all participants were given diaries to note the incidence and severity of symptoms and requested to report any adverse effects.

### 2.6. Statistical Analysis

Data were analysed using the Statistical Package for the Social Sciences software (SPSS 19) for Windows. The antioxidant activities were tested in triplicate in at least three different experiments, and their results were presented as mean ± SD. The differences in laboratory parameters between the tested condition at baseline and after 4th and 8th week were assessed using a two-way ANOVA with Fisher's Least Significant Difference (LSD) as the post hoc test. A difference was considered statistically significant when the *p* value was less than 0.05.

## 3. Results

### 3.1. Characteristics of TSP

The optimal brewing times for TSP tea were determined with respect to the highest antioxidant activities observed with acceptable sensory parameters. According to the results (Table 1), no significant differences in sensory scores for infusion colour, aroma, test, and overall acceptability were found among TSP teas brewed for 1, 3, and 5 min at 95°C (*p* < 0.05). Antioxidant capacity assessment of TSP tea showed that all samples were able to quench ABTS and DPPH radicals. TSP tea brewed for 3 and 5 min yielded significantly higher free radical scavenging activities than TSP tea infused for 1 min. Brewing of TSP tea for 3 min at 95°C was chosen for further study because of its remarkable free radical scavenging capacity and slightly high sensory scores for infusion colour and aroma.

The yield of pale yellow oils obtained from TSP was 0.52% (v/w). The constituents of TSP oil include 1,8-cineole (27.7 ± 0.3 mg/mL), alpha-terpineol (39.7 ± 0.4 mg/mL), linalool (1.3 ± 0.1 mg/mL), and methyl eugenol (7.7 ± 0.4 mg/mL). Brewed TSP tea used in this study possessed total phenolic content (expressed as gallic acid) equivalent to 3092.79 ± 113.88 *μ*g/g of dried extract and total flavonoid content (expressed as catechin) equivalent to 131.25 ± 3.46 mg/g of dried extract.

### 3.2. Health Benefits of TSP Consumption on Overweight Volunteers

**Figure 2:**
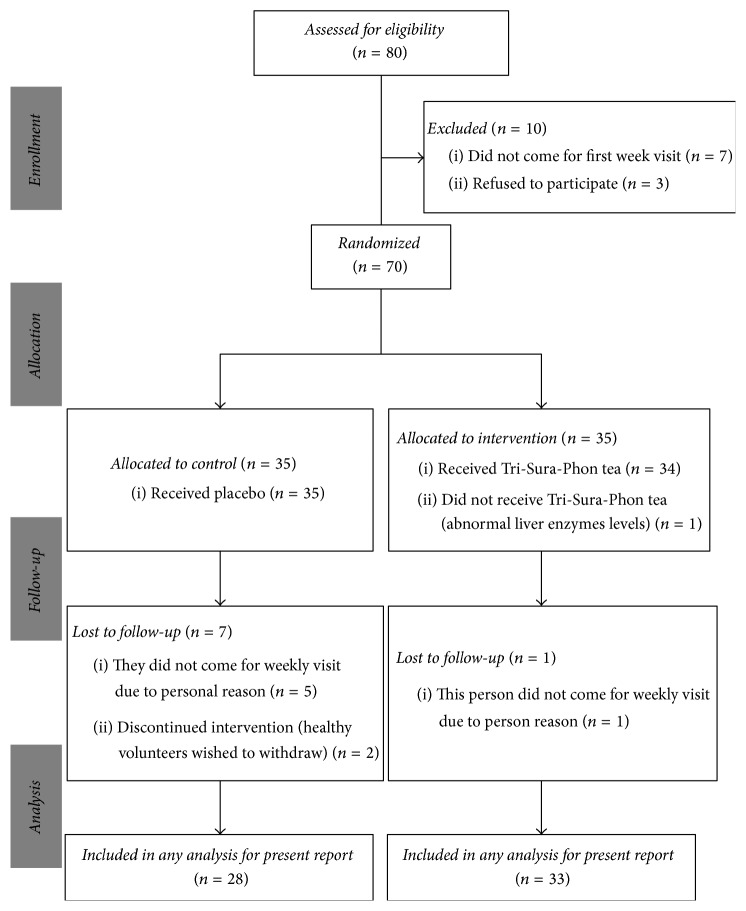
CONSORT diagram reflecting flow of study participants through the study.

Of the 80 participants enrolled in this study, 61 completed the trial, and the following cases were excluded from the intervention: three volunteers refused to participate, seven did not attend the first-week visit, one showed abnormal liver function in tests, and eight were lost to follow-ups. The CONSORT flowchart of the progress of the participants throughout the study is shown in Figure 2. There were no significant differences in baseline parameters between the two groups (Table 2; *p* ≥ 0.05).

The average triglyceride level in the TSP group decreased significantly from 147.6 mg/dL to 125.6 (at the 4th week; *p* = 0.000) and 96.1 mg/dL (at the 8th week; *p* = 0.001) compared with that at the baseline (Table 2). In the TSP group, significant decreases in the average fasting levels of total cholesterol and LDL were additionally observed after 8 weeks of TSP treatment compared with that at the baseline (*p* = 0.013 for total cholesterol and *p* = 0.017 for LDL). The average HDL level in the TSP group at the beginning of the study was 65.2 mg/dL, and it increased significantly (*p* = 0.005) to 72.4 mg/dL after 8 weeks of TSP treatment. No significant differences were observed in the placebo group before and after the intake in terms of total cholesterol, LDL, and HDL levels, but the average triglyceride levels in the placebo group decreased significantly (*p* = 0.002 at the 4th week). Moreover, changes of the cholesterol, LDL, and HDL levels following 8-week consumption of TSP were significantly higher than those following placebo treatment (*p* = 0.005; Table 3). No significant differences were found in terms of BMI and insulin levels; however, the FBG levels increased significantly in both TSP and placebo groups when compared between the baseline and endpoint.

No adverse effects were reported during the study. Neither the consumption of TSP infusion nor the placebo caused significant changes in the levels of direct bilirubin, AST, ALP, and GGT (Table 2). The TSP infusion lowered the blood levels of albumin (at the 8th week; *p* = 0.000) and ALP (at the 8th week; *p* = 0.000) significantly. In the placebo group, albumin level (*p* = 0.008 at the 4th week) decreased compared to that at the baseline, whereas total bilirubin and albumin levels decreased when compared to the levels between the 4th and 8th week (*p* = 0.031 for total bilirubin and *p* = 0.000 for albumin).

## 4. Discussion

Traditionally, Thai herbal medicines are applied as a formulated decoction, which includes a specific combination of different medicinal plants. These polyherbal medicines have been clinically used for thousands of years and currently play an indispensable role in the prevention and treatment of diseases. Based on the fact that the traditional Thai formulation was used as a rejuvenator and recorded in the Thai Pharmaceutical Textbook, the beneficial effects of TSP were determined in healthy overweight volunteers owing to its safety and efficacy.

The present results showed that an 8-week period of TSP tea drinking (2 cups per day) safely improves serum lipid profiles in overweight or obese volunteers. The consumption of this polyherbal tea, which contains antioxidant chemicals, was accountable for the decrease in highly atherogenic LDL-C levels and increase in HDL-C levels. LDL-C particles are more easily oxidized, are less readily cleared, and have been linked to an increased risk of atherosclerosis, whereas HDL-C particles provide protection against the formation of atherosclerotic plaques [23]. Therefore, evidence from our study is positively relevant to the control of dyslipidemia, which has been frequently observed in patients who are obese or have diabetes mellitus. No significant changes were found in the BMI of overweight volunteers following TSP tea consumption, which was in agreement with a 12-week period of green tea extract consumption in obese women [24]. However, obese women who were treated with green tea extract that contains highly antioxidant polyphenols showed significant reductions in total cholesterol, triglyceride, and LDL-C levels and a marked increase in HDL-C levels [7, 24].

An increasing number of studies confirm that increased systemic oxidative stress in overweight or obese patients directly impacts the insulin sensitivity of metabolic organs, promotes inflammation, alters lipid metabolism, and causes endothelial dysfunction [4]. Consuming antioxidative agents such as vitamin C, vitamin E, and selenium can reduce oxidative stress, and improve liver functions in obese patients [25], and reverse endothelial dysfunction in patients with coronary risk factors [26]. Therefore, lowering oxidative stress to prevent obesity-associated disorders establishes an interesting goal. The antioxidant mechanisms of medicinal plants may be controlled by stimulating endogenous antioxidant defence systems, scavenging reactive species, or chelating transition metals, thus stopping the progressive autooxidative damage [27]. In our current study, TSP showed effective antioxidant activity in part as a free radical scavenger, which could be especially beneficial for obesity-associated low-grade inflammation.

TSP infusion is rich in flavonoids and polyphenols, which seem to be the key factors responsible for the antioxidant activities of TSP and demonstrate beneficial effects on serum lipid profiles. For example, polyphenols in green tea extract affect the absorption of cholesterol and glucose in the small intestine [28, 29]. Phytosterols, curcumin, and (−)-epigallocatechin gallate have been found to affect lipid metabolism by reducing the absorption of cholesterol as well as gluconeogenesis and lipogenesis via activation of AMP-activated protein kinase or peroxisome proliferator-activated receptors [30]. Among the herbal components tested, notable antioxidant activity and phytochemical constituents were observed for* C. bejolghota*. This plant is widely used in Thai cuisine to add an aromatic flavour. Although the major active components of* C. bejolghota* had never been previously studied, preliminary phytochemical tests showed that methanol and water extracts prepared from* C. bejolghota* bark contained glycosides, saponins, tannins, and phenolic compounds [17]. In a similar study, Settharaksa et al. (2012) reported that* C. bejolghota* possesses the highest total phenolic content and ferric reducing antioxidant power among the tested ingredients in Kua-Khling curry paste, which is used in Thai cuisine [31]. It should be further noted that the methanol extract of* C. bejolghota* bark significantly reduced cholesterol, triglyceride, and LDL-C levels and decreased HDL-C levels in streptozotocin- (STZ-) induced diabetic rats [17]. In addition, the extract has been found to reduce lipid peroxidation and improve the level of catalase and glutathione in their livers. However, there is no available report for the hypolipidemic and antioxidant effects of* C. parthenoxylon*. Jia et al. (2009) found that the water-soluble polyphenolic oligomers from* C. parthenoxylon* have hypoglycemic effects in both normal and STZ-induced diabetic rats [16]. Our results showed that the antioxidant activities and contents of total phenolics and flavonoids of* A. crassna* were less than those of the extracts of* C. parthenoxylon* and* C. bejolghota*. Essential oils extracted from* A. crassna* possess antioxidant and anticancer activities, but a recent study revealed that feeding these essential oils to mice did not affect the triglyceride, LDL-C, and HDL-C levels [18]. It should be noted that 1,8-cineole [32], linalool [33], and methyl eugenol [34], which are components of TSP essential oils, have been reported to positively affect the serum lipid profile in in vivo models. Based on these results, it may be hypothesized that the beneficial effects of TSP tea observed in healthy overweight volunteers may be primarily caused by* C. parthenoxylon* and* C. bejolghota*. However, studies investigating the active constituents and mechanisms that mediate lipophilic effects of* Tri-Sura-Phon* tea and the influence of tea consumption on human oxidative status need to be pursued.

The standardization of polyherbal formulation according to its active components, as well as the use of a placebo having no effect on serum lipid profile, can be cited as the strengths of the current study. The limitations of the study include small sample size with a narrow range of participant ages and BMI as well as the use of healthy subjects. Although, having a placebo arm is the strength of the present study, there are differences in the appearance of colour, odour, and taste between the placebo and TSP infusion should be noted as the limitation. Lack of qualitative information on the diet and exercise as well as quantitative data on energy and nutrient intake of the participants can be also mentioned as the limitation of this study.

## 5. Conclusions

In conclusion, our findings showed that the total cholesterol, triglyceride, LDL-C, and HDL-C levels in overweight volunteers were affected by taking* Tri-Sura-Phon* tea, which might be a useful adjunctive remedy for overweight patients. The observed health benefits may, at least in part, be associated with the free radical scavenging properties of the tea. Further trials on the long-term safety and efficacy of this tea in vivo and in the treatment of borderline hyperlipidemia are currently being investigated by our research group.

## Figures and Tables

**Table 1:** Antioxidant capacities and consumer acceptability of Tri-Sura-Phon teas with different brewing times.

	Brewing time (min)		
	1	3	5
Antioxidant capacities^*∗*^ (% ± SD)			
(i) DPPH radical scavenging capacity	85.69 ± 1.70^a^	89.45 ± 0.63^b^	88.49 ± 0.24^b^
(ii) ABTS radical scavenging capacity	84.52 ± 4.10^a^	99.57 ± 0.00^b^	100.00 ± 0.00^b^
Sensory analysis			
(i) Colour	6.60 ± 1.77^a^	6.63 ± 1.77^a^	6.43 ± 1.87^a^
(ii) Aroma	5.33 ± 2.17^a^	5.53 ± 2.11^a^	5.23 ± 2.11^a^
(iii) Taste	4.73 ± 2.02^a^	5.23 ± 2.19^a^	5.30 ± 2.89^a^
(iv) Overall acceptability	4.77 ± 2.13^a^	5.30 ± 2.04^a^	5.30 ± 1.97^a^

^*∗*^The tested teas exhibited DPPH and ABTS radical scavenging activities at concentration of 1% (v/v).

^a,b^Means bearing different superscript letters in the same row are significantly different (*p* < 0.05).

**Table 2:** Effect of Tri-Sura-Phon tea drinking on body mass index, serum lipid profile, glucose and insulin levels, and liver function of healthy overweight volunteers.

Parameters^*∗*^	Tested group	Mean (SD)		
		Baseline	4th week	8th week
BMI (kg/m^2^)	Tri-Sura-Phon	27.7 (4.5)	27.6 (4.5)	27.6 (4.5)
	Placebo	28.3 (4.0)	28.0 (4.2)	28.2 (4.1)
Total cholesterol (mg/dL)	Tri-Sura-Phon	223.7 (37.6)	213.5 (29.2)	201.3 (36.2)^B^
	Placebo	210.5 (33.0)	213.6 (37.2)	211.9 (42.6)
Triglycerides (mg/dL)	Tri-Sura-Phon	147.5 (74.3)^3^	79.3 (24.2)^A,1,2^	103.4 (40.2)^B,1^
	Placebo	142.4 (75.9)	125.6 (61.5)	96.1 (37.5)^B,C^
Low density lipoprotein (mg/dL)	Tri-Sura-Phon	130.5 (40.5)	129.7 (30.6)	108.5 (37.6)^B,C,3^
	Placebo	116.2 (35.5)	125.0 (33.7)	131.7 (44.0)
High density lipoprotein (mg/dL)	Tri-Sura-Phon	63.4 (12.8)	68.0 (14.1)^3^	72.4 (13.4)^B,1,3 ^
	Placebo	65.8 (10.0)	67.6 (11.4)	60.9 (13.6)^C^
FBG (mg/dL)	Tri-Sura-Phon	76.2 (6.1)^1,2,3^	84.1 (7.9)^A,2,3^	84.3 (8.4)^B,2,3^
	Placebo	81.5 (12.0)	91.4 (12.8)^A^	91.2 (9.0)^B^
Insulin level (uU/mL)	Tri-Sura-Phon	8.9 (4.1)	8.3 (3.5)^3^	9.9 (5.0)
	Placebo	10.9 (7.0)	8.7 (4.2)	10.9 (7.0)
Albumin (gm/dL)	Tri-Sura-Phon	4.6 (0.5)^2^	4.5 (0.5)^2^	4.1 (0.5)^B,C,1,3^
	Placebo	4.5 (0.5)	4.1 (0.4)^A^	4.6 (0.5)^C^
Total bilirubin (mg/dL)	Tri-Sura-Phon	0.7 (0.4)	0.9 (0.3)^3^	0.8 (0.4)
	Placebo	0.8 (0.5)	0.9 (0.4)	0.6 (0.5)^C^
Direct bilirubin (mg/dL)	Tri-Sura-Phon	0.0 (0.0)	0.0 (0.0)	0.0 (0.0)
	Placebo	0.0 (0.2)	0.0 (0.0)	0.0 (0.0)
AST (U/L)	Tri-Sura-Phon	20.1 (5.6)^2,3^	22.6 (6.8)	19.8 (4.3)^2,3^
	Placebo	22.7 (7.1)	24.2 (5.1)	24.3 (13.2)
ALT (U/L)	Tri-Sura-Phon	21.1 (14.0)^3^	20.8 (13.4)^3^	18.7 (13.5)^3^
	Placebo	22.5 (11.6)	20.7 (11.1)	30.5 (26.5)^C^
ALP (U/L)	Tri-Sura-Phon	78.1 (20.1)	76.0 (19.5)	69.6 (21.3)
	Placebo	77.5 (15.7)	74.8 (18.3)	74.5 (17.0)
GGT (U/L)	Tri-Sura-Phon	25.0 (15.7)	25.7 (16.0)	25.1 (17.7)
	Placebo	25.5 (8.6)	23.0 (8.3)	26.0 (12.2)

Uppercase superscript letters indicate the significant differences in parameters between the baseline and week 4 (A) or the baseline and week 8 (B) or weeks 4 and 8 (C) within the treatment.

Superscript numbers indicate the significant differences between the parameters at baseline (1), week 4 (2), or week 8 (3) among the treatments.

^*∗*^BMI = body mass index, FBG = fasting blood glucose, AST = aspartate aminotransferase, ALT = alanine aminotransferase, ALP = alkaline phosphatase, and GGT = gamma-glutamyl transpeptidase.

**Table 3:** Changes in clinical laboratory parameters after the consumption of Tri-Sura-Phon tea.

Parameters^*∗∗*^	Tested groups	Changes of evaluated values (SD)^*∗*^		
		wk. 0 *versus* wk. 4th	wk. 0 *versus *wk. 8th	wk. 4th *versus* wk. 8th
BMI (kg/m^2^)	Tri-Sura-Phon	0.1 (0.4)^3^	0.1 (0.5)^3^	0.0 (0.3)^1^
	Placebo	0.3 (0.5)	0.1 (0.7)	−0.2 (0.5)^A,B^
Total cholesterol (mg/dL)	Tri-Sura-Phon	10.1 (34.0)	22.4 (42.4)^1,2,3^	12.2 (22.3)
	Placebo	−3.2 (33.2)	−1.5 (29.8)	1.7 (29.6)
Triglycerides (mg/dL)	Tri-Sura-Phon	68.2 (79.3)^1,3^	44.1 (80.8)	−24.1 (47.3)^A,B,1,2,3^
	Placebo	16.8 (97.7)	46.3 (76.5)	29.5 (57.7)
Low density lipoprotein (mg/dL)	Tri-Sura-Phon	0.9 (38.8)	22.1 (49.2)^A,1,2,3^	21.18 (26.7)^A,1,2,3^
	Placebo	−8.9 (36.0)	−15.5 (38.9)	−6.7 (35.2)
High density lipoprotein (mg/dL)	Tri-Sura-Phon	−4.6 (13.3)^2,3^	−9.0 (15.3)^1,2,3^	−4.4 (11.8)^2,3^
	Placebo	−1.8 (12.6)	4.9 (12.7)	6.7 (14.9)^A^
FBG (mg/dL)	Tri-Sura-Phon	−7.8 (7.8)^3^	8.1 (7.0)^3^	−0.3 (7.2)^A,B,1,2^
	Placebo	−10.0 (7.7)	−9.7 (8.1)	0.2 (8.3)^A,B^
Insulin level (uU/mL)	Tri-Sura-Phon	0.5 (4.0)	−1.0 (4.6)	−1.7 (4.4)
	Placebo	−0.2 (5.9)	−2.4 (8.2)	−2.0 (7.7)
Albumin (gm/dL)	Tri-Sura-Phon	0.1 (0.4)^3^	0.4 (0.7)^A,2,3^	0.1 (0.5)^B,2^
	Placebo	0.2 (0.5)	0.0 (0.2)	−0.3 (0.5)^A^
Total bilirubin (mg/dL)	Tri-Sura-Phon	0.0 (0.2)	0.0 (0.0)^2,3^	0.1 (0.3)
	Placebo	0.0 (0.2)	0.1 (0.3)^A^	0.1 (0.4)^A^
Direct bilirubin (mg/dL)	Tri-Sura-Phon	0.0 (0.0)	0.0 (0.0)	0.0 (0.0)
	Placebo	0.0 (0.2)	0.0 (0.2)	0.0 (0.0)
AST (U/L)	Tri-Sura-Phon	−2.5 (7.2)	0.2 (5.3)	2.8 (5.2)^A,1,2^
	Placebo	−1.5 (7.4)	−1.6 (9.8)	−0.1 (13.4)
ALT (U/L)	Tri-Sura-Phon	0.3 (10.2)^2,3^	2.3 (11.4)^2,3^	2.0 (7.7)^2,3^
	Placebo	1.8 (8.9)	−7.9 (24.1)^A^	−9.7 (23.9)^A^
ALP (U/L)	Tri-Sura-Phon	2.1 (9.3)	8.6 (12.4)^A,3^	6.4 (11.2)
	Placebo	2.7 (15.1)	3.0 (11.4)	0.36 (16.8)
GGT (U/L)	Tri-Sura-Phon	−0.7 (9.4)	−0.1 (8.7)	0.6 (4.1)
	Placebo	2.5 (5.1)	−0.5 (8.5)	−3.0 (9.6)^A^

Uppercase superscript letters indicate significant differences in changes of parameters between baseline and week 4 (A) or baseline and week 8 (B) or weeks 4 and 8 (C) in the same treatment.

Superscript numbers indicate significant differences in changes of parameters between baseline and week 4 (1) or baseline and week 8 (2) or weeks 4 and 8 (3) among the treatments.

^*∗*^Negative values correspond to increases from baseline and positive values correspond to decreases from baseline.

^*∗∗*^BMI = body mass index, FBG = fasting blood glucose, AST = aspartate aminotransferase, ALT = alanine aminotransferase, ALP = alkaline phosphatase, and GGT = gamma-glutamyl transpeptidase.
